# Supplementary figures and images for: Cloning and functional verification of Geraniol-10-Hydroxylase gene in Lonicera japonica
Source: PeerJ. 2025 Jan 13;13:e18832. doi: 10.7717/peerj.18832 (PMC11737341; doi:10.7717/peerj.18832)

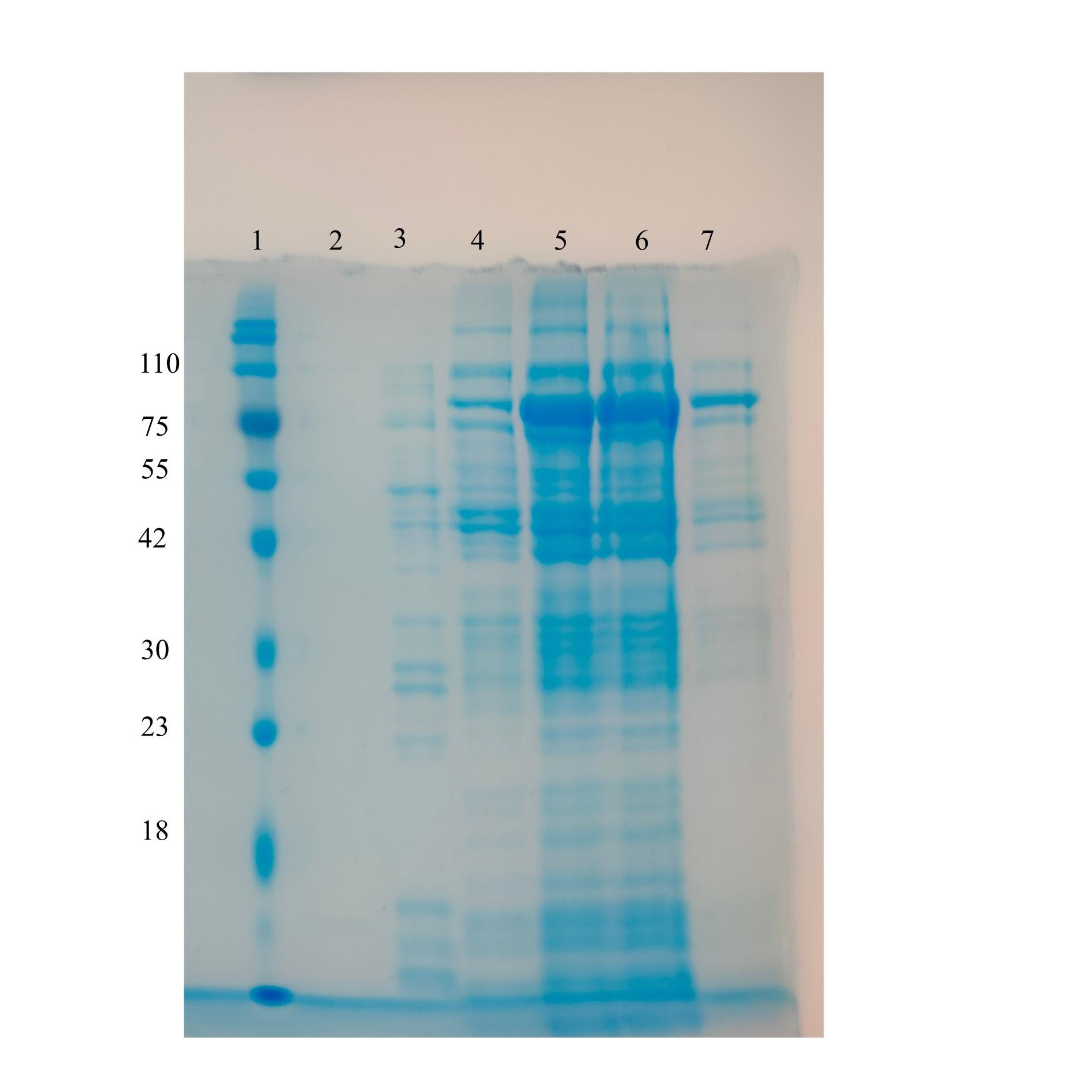

Supplement: Supplemental Information 1 [file peerj-13-18832-s001.jpg]

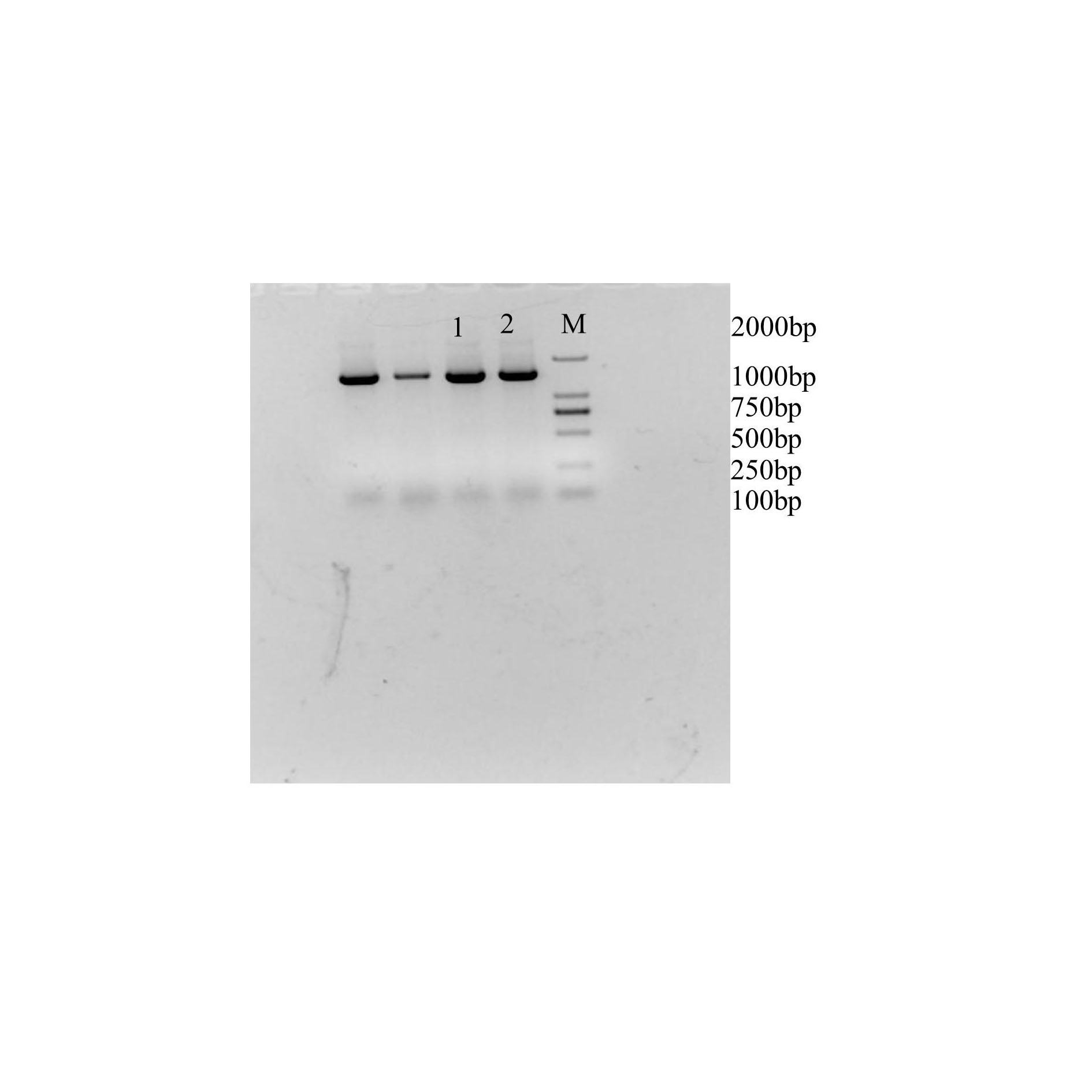

Supplement: Supplemental Information 2 — Amplification using Lonicera japonica cDNA as a template. [file peerj-13-18832-s002.jpg]
